# Physiological and Transcriptomic Analysis of *Arabidopsis thaliana* Responses to Ailanthone, a Potential Bio-Herbicide

**DOI:** 10.3390/ijms231911854

**Published:** 2022-10-06

**Authors:** Chantz Allen Hopson, Purushothaman Natarajan, Suhas Shinde, Arjun Ojha Kshetry, Krishna Reddy Challa, Armando Pacheco Valenciana, Padma Nimmakayala, Umesh K. Reddy

**Affiliations:** Department of Biology, Gus R. Douglass Institute, West Virginia State University, Institute, WV 25112, USA

**Keywords:** ailanthone, herbicides, sustainability, agriculture, transcriptome, Arabidopsis

## Abstract

Many plants naturally synthesize and secrete secondary metabolites that exert an allelopathic effect, offering compelling alternatives to chemical herbicides. These natural herbicides are highly important for sustainable agricultural practices. Ailanthone is the chemical responsible for the herbicidal effect of *Ailanthus altissima*, or “tree of heaven”. The molecular studies involving ailanthone’s effect on plant growth are limited. In the current study, we combined whole-transcriptome and physiology analysis of three *Arabidopsis thaliana* ecotypes treated with ailanthone to identify the effect of this allelopathic chemical on genes and plant growth. Our physiology results showed 50% reduced root growth, high proline accumulation, and high reactive-oxygen-species accumulation in response to ailanthone stress. Deep transcriptome analysis revealed 528, 473, and 482 statistically significant differentially expressed genes for Col-0, Cvi-0, and U112-3 under ailanthone stress, including 131 genes shared among the three accessions. The common genes included 82 upregulated and 42 downregulated genes and varied in expression at least twofold. The study also revealed that 34 of the 131 genes had a similar expression pattern when Arabidopsis seedlings were subjected to other herbicides. Differentially expressed genes significantly induced in response to ailanthone included *DTXL1*, *DTX1*, *ABCC3*, *NDB4*, *UGT74E2*, and *AZI1*. Pathways of stress, development and hormone metabolism were significantly altered under ailanthone stress. These results suggest that ailanthone triggers a significant stress response in multiple pathways similar to other herbicides.

## 1. Introduction

Allelopathy refers to the chemical inhibition of one plant by another via the secretion of a chemical into the environment, which acts as a growth inhibitor. The chemicals responsible for this inhibition are known as allelochemicals. Ailanthone is one such allelochemical produced by *Ailanthus altissima*, commonly known as the tree of heaven. This chemical is a quassinoid that suppresses the growth and germination of other plants around the tree by secretion through various plant organs. The highest concentration is in the roots and the bark, but the chemical can be found in all plant parts, including the leaves and fruit [1]. The tree of heaven is an invasive species originating in Southeast Asia. It has caused many environmental issues due to its ability to reproduce rapidly and inhibit the growth of native plants in the area. In many cases, this chemical is a natural herbicide and will likely be used as a bioherbicide.

Synthetic herbicides are the dominant means of controlling weed pests in agriculture today. These herbicides become less effective over time because they have a particular mode of action that allows the weed pests to quickly build up resistance [2]. This requires more potent synthetic herbicides to prevent weed pests from taking over crop lands and causing significant losses in overall yield. The reduction in yield may be attributed to the competition for light, nutrients, and water between weed pests and crop plants. These synthetic herbicides may be helpful and have been the primary weed/pest control method for decades. However, they are harmful to the environment and humans, polluting waterways through leaching and crop run-off, damaging the microbiome with toxic chemicals that subsist in the soil for long periods, and causing many sicknesses and cancers in humans and animals [2]. Because ailanthone is a natural plant product, it will degrade faster in the environment than its synthetic counterparts [1].

*A. altissima*, commonly known as the tree of heaven, is a deciduous tree species in the family Simaroubaceae native to Southeast Asia. It is a highly invasive species, widespread in many countries, especially in Europe and North America, where the tree was introduced as an ornamental species. The tree of heaven reproduces rapidly from both seeds and clonally via root shoots, with high seed production of up 325,000 samaras in an eight-year-old tree. Stem growth is highly rapid, and *A. altissima* is believed to be one of the fastest-growing trees in North America. As an invasive species, the tree of heaven has many adverse effects on the native biodiversity because of altered soil properties, rapid growth and reproduction, and its very unpleasant smell and allergenic properties. Its allelopathic effects can inhibit growth and kill plants around the tree base. However, *A. altissima* does have some ecological benefits, such as its use in land reclamation and reforestation, in paper production, as timber, and for its medicinal properties [3]. Many plants use allelochemicals to out-compete other higher plants for survival and reproduction. Allelopathy allows invasive species such as *A. altissima* to quickly take over and out-compete many native plant ecosystems by inhibiting the growth of other plants. Allelopathy can also have a negative effect on the plants that produce them; examples include corn, sugarcane, and rice [4]. Ailanthone has shown great potential as a bioherbicide, but its mode of action and effects on other organisms are still unknown [5]. 

RNA sequencing is a high-throughput sequencing method that can decode the expression profile of all the RNA populations in a cell. Hence, in the present study, we used physiological and transcriptomic approaches to study the effect of ailanthone in a model plant, *Arabidopsis thaliana*. We studied the effect of ailanthone in three different ecotypes of *A. thaliana*: Col-0, Cvi-0, and U112-3.

## 2. Results

### 2.1. Dose–Response Effect of Ailanthone on Primary Root Length (PRL)

**Figure 1:**
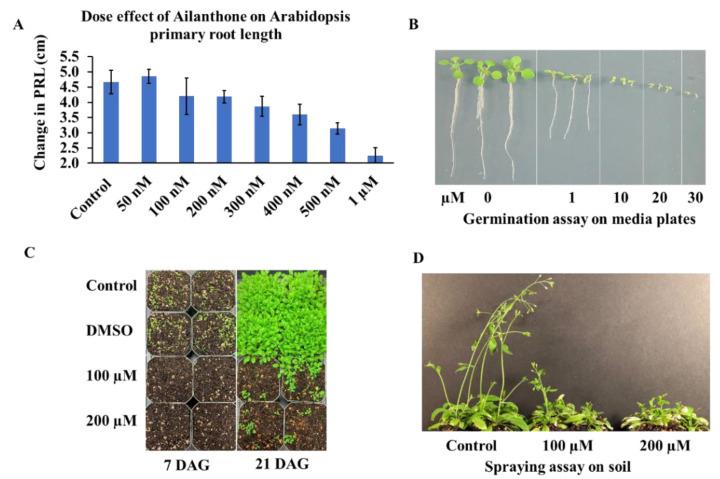
Effect of ailanthone on Arabidopsis primary root growth and seed germination. Seven-day-old Col-0 seedlings were transferred to media plates supplemented with different ailanthone concentrations. Change in root growth was quantified on day 7 after the onset of the treatment (*p* < 0.001) (**A**). Arabidopsis seeds were germinated in vitro on growth media supplemented with 1–30 µM ailanthone and representative germinated seedling images are presented (**B**,**C**) Col-0 seeds were germinated and grown on ailanthone-treated soil for 7 and 21 days. The top soil was sprayed with or without ailanthone (DMSO as a mock and control, no DMSO). Representative images were taken at 7 and 21 days after germination (DAG). (**D**) Seedlings were grown to maturity shown in (**C**). Representative photos were taken at 50 DAG.

Ailanthone treatments significantly affected the primary root growth of *A. thaliana* seedlings. To determine the effective dose of ailanthone on Arabidopsis root growth, we treated 8-day-old Col-0 seedlings with ailanthone concentrations ranging from 50 nM to 1 µM. Higher levels of ailanthone significantly inhibited the PRL (Figure 1A). The lowest ailanthone concentrations (50, 100, and 200 nM) did not significantly affect the PRL. In contrast, 300 nM ailanthone induced growth reduction by ~17%, which reached 22% inhibition at 400 nM ailanthone as compared with the control (i.e., mock DMSO) (Figure 1A). Finally, the highest concentrations of ailanthone assayed (500 nM and 1 µM) induced ~33% to 53% PRL inhibition. Therefore, ailanthone induced PRL inhibition dose-dependently.

### 2.2. Ailanthone Induces Plant Growth Inhibition Dose-Dependently

To elucidate the effects of ailanthone on plant growth, we germinated *A. thaliana* (Col-0 wild type) seeds in the presence of different ailanthone concentrations (0, 1, 10, 20, and 30 µM). Col-0 seeds showed 100% germination in all treatments when observed on the MS agar media plates; however, higher levels of ailanthone (10, 20, and 30 µM) completely inhibited seedling growth (Figure 1B). The seedlings grew in the presence of 1 µM ailanthone, but growth was ~50% less than in seedlings grown under control conditions (Figure 1B). Therefore, ailanthone did not affect seed germination but overall inhibited plant growth. Similarly, we conducted soil experiments by spraying higher concentrations of ailanthone (100 and 200 µM) after sowing Col-0 seeds. The seeds showed significant germination in control and mock (DMSO) treatment (Figure 1C). However, seedling numbers in ailanthone-treated pots were significantly reduced (Figure 1C). We let plants grow in pots to maturity. Ailanthone-treated plants showed highly stunted growth and development (Figure 1D), which suggests that ailanthone might have a potent herbicidal effect on plant growth at the foliar stage instead of affecting seed germination. 

### 2.3. Effect of Ailanthone on Arabidopsis Root Growth

**Figure 2:**
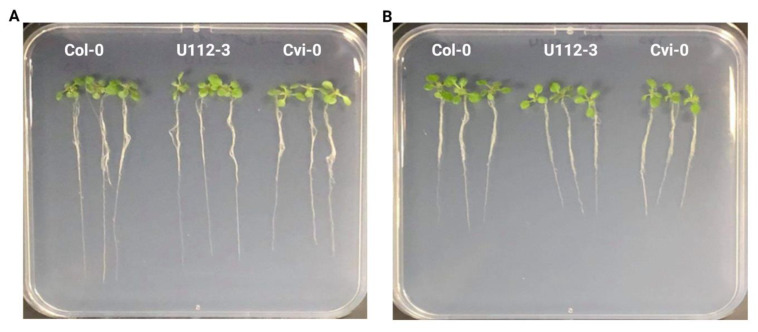
Effect of ailanthone on the Arabidopsis root growth in control (**A**) and treatment (**B**) conditions across three ecotypes, Col-0, U112-3, and Cvi-0.

**Figure 3:**
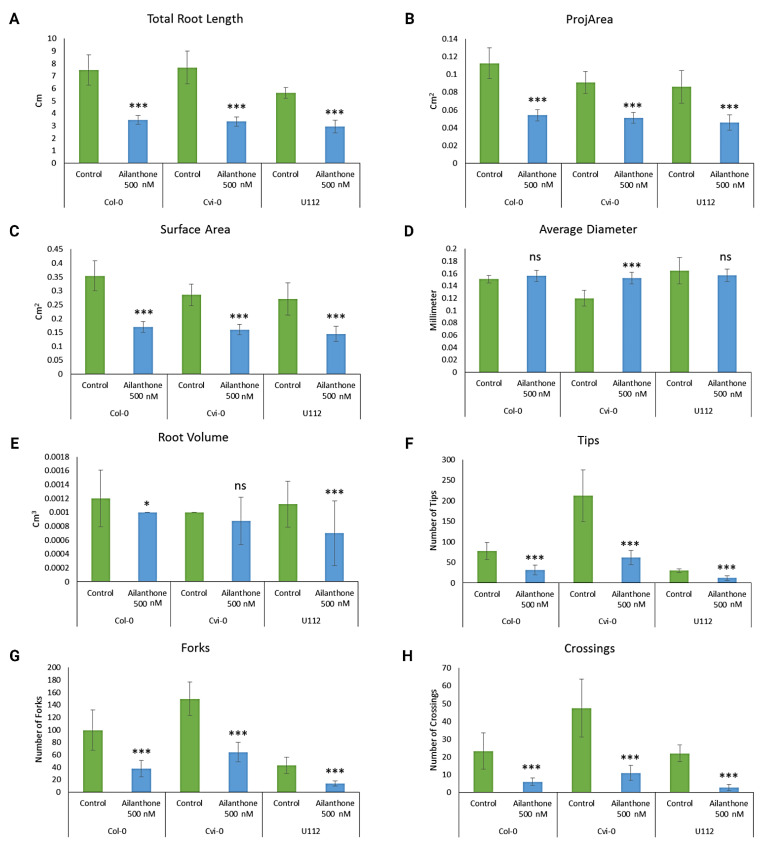
Effect of ailanthone on (**A**) primary root length, (**B**) root projected area, (**C**) root surface area, (**D**) root diameter, (**E**) root volume, (**F**) number of root tips, (**G**) number of root forks and (**H**) number of root crossings of the three ecotypes in control and treatment conditions. Data are mean SEM. *** *p* < 0.001; * *p* < 0.05; ns: non-significant.

Col-0, Cvi-0, and U112-3 seeds were surface-sterilized and grown with 0.5 µM ailanthone along with mock controls, and the seedling response was checked by analyzing overall root length, root tips, root volume, forks, and crossings. The root-trait data were recorded on day 12 (5 days after being introduced to control and treatment plates) and were recorded by using the WinRhizo software. The difference in growth phenotypes between control and treatment plates of Col-0, U112-3, and Cvi-0 seedlings is shown in Figure 2 and Figure 3.

The overall primary root length was ~7.5 cm for the Col-0 control but ~4 cm for ailanthone-treated Col-0 plants; Cvi-0 showed similar results to Col-0 in both control and treatment conditions (Figure 3A). U112-3 showed slightly smaller overall root length in both control (5 cm) and treatment (3 cm) conditions. Each ecotype showed ~50% primary root growth reduction in response to ailanthone treatment. The average projected area of Col-0, Cvi-0, and U112-3 was decreased by ~50% in the treatment versus control condition (Figure 3B). A similar result was seen in the average surface area, which decreased by ~50% in the treatment versus control condition (Figure 3C). The average root diameter was slightly increased for Cvi-0 in the treatment condition, but the Col-o and U112-3 root diameter was not changed significantly in the treatment versus control condition (Figure 3D). Average root volume decreased by varying amounts in the treatment versus control condition for the three ecotypes, with U112-3 the most heavily effected, Cvi-0 the least effected, and Col-0 having an intermediate effect (Figure 3E). Control-treated Cvi-0 plants exhibited a higher number of root tips than Col-0 and U112-3 plants (Figure 3F). As expected with regular herbicide treatments, the 0.5-µM ailanthone-treated Cvi-0 root tip number was significantly reduced from 212 to 79, a reduction of 62%. However, ailanthone-treated Col-0 and U112-3 seedlings also displayed a reduced total number of root tips but to a lesser extent (50%) (Figure 3F), which suggests that Cvi-0 plants are more sensitive to ailanthone than Col-0 and U112-3 plants. Root forks and crossovers also showed similar results of ailanthone treatment in Col-0, Cvi-0, and U112-3 (Figure 3G,H). Col-0, U112-3, and Cvi-0 had an even more significant reduction than the control. These results suggest that ailanthone inhibits root growth, thereby inducing stress on plant growth and development.

### 2.4. Ailanthone Induces Proline Biosynthesis

**Figure 4:**
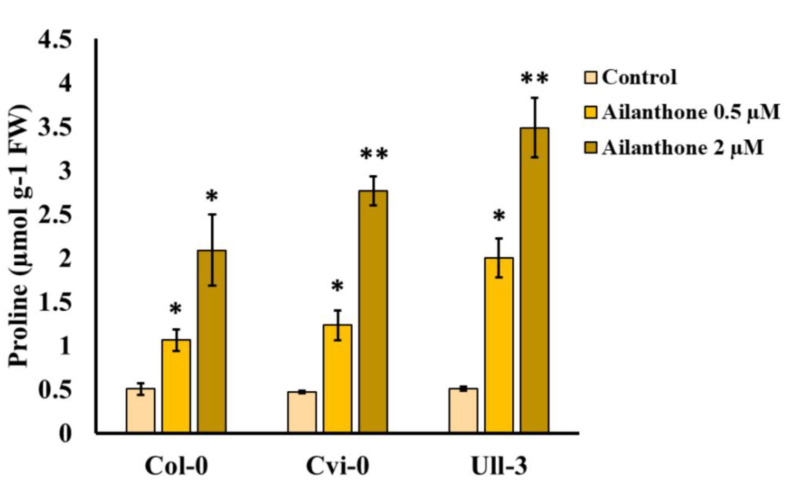
Ailanthone treatment induces proline content. Quantifying proline content in 12-day-old MS-grown Col-0, Cvi-0, and U112-3 seedlings shifted to the MS medium supplemented with mock (control), 0.5 and 2 µM ailanthone for 1 week. Sample number, *n* = 3; error bars, SD; * indicates *p* < 0.05, ** indicates *p* < 0.01 (unpaired Student’s *t*-test).

Proline biosynthesis has been linked to the oxidative pentose phosphate (OPP) pathway and glutamate-glutamine metabolism [6]. Several stress responses induce proline content; therefore, its quantification serves as a marker for plant stress. To check the ailanthone effect on Arabidopsis ecotypes (Col-0, Cvi-0, and U112-3), we treated the 12-day-old seedlings with 0.5 and 2 µM ailanthone for 1 week to overcome the continuous ailanthone effect on plant growth and analyzed proline content in 18-day-old seedlings. Consistent with the ailanthone-induced substantial growth retardation, proline content was significantly increased two- to fourfold in all three ecotypes. Mock-treated Col-0 seedling proline content was measured at 0.5 ± 0.07 µmol g-1 fresh weight (FW), and it was increased to 1.0 ± 0.12 and 2.0 ± 0.14 µmol g-1 FW upon 0.5 and 2 µM ailanthone treatments, respectively (Figure 4). Similarly, Cvi-0 and U112-3 seedlings showed two- and fourfold increases in proline accumulation with 0.5 and 2 µM ailanthone treatments, which suggests that ailanthone induces a stress response in plants similar to other herbicides and inhibits plant growth.

### 2.5. Ailanthone Accumulates Excessive ROS 

**Figure 5:**
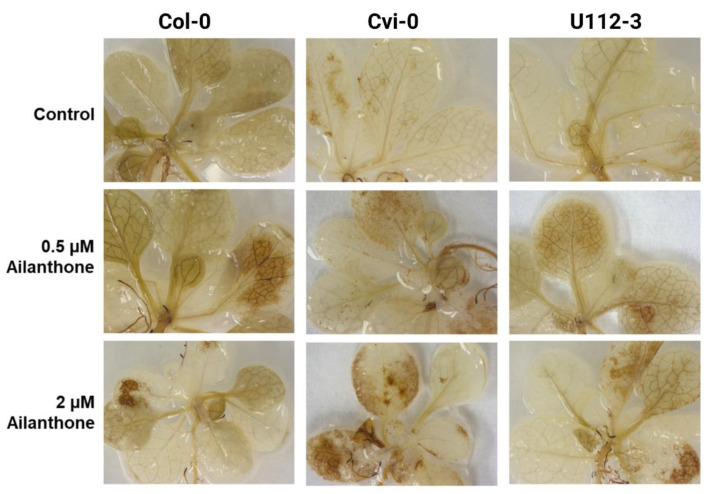
Ailanthone-treated seedlings accumulate excessive ROS. DAB staining of MS-grown 12-day-old Col-0, Cvi-0, and U112-3 seedlings shifted to the MS medium supplemented with mock (control), 0.5 and 2 µM ailanthone for a week.

Similar to proline accumulation, ROS is also strongly induced by biotic and abiotic stresses. We further aimed to check the ailanthone effect on ROS accumulation in Col-0, Cvi-0, and U112-3 by DAB staining. Rosettes that were 18 days old and treated with 0.5 and 2 µM ailanthone were analyzed with DAB. Col-0, Cvi-0, and U112-3 leaves stained more intensively when plants were grown on 0.5 and 2 µM ailanthone as compared with control leaves (Figure 5). These results suggest that ailanthone induces ROS accumulation and a stress response in plants.

### 2.6. Transcriptome Analysis

A total of 178,639,612, 187,148,924, and 178,521,414 raw reads were generated for Col-0, Cvi-0, and U112-3, respectively. The raw reads were subjected to stringent quality filtering by using the Trimmimatic tool, which resulted in 166,058,758, 174,167,848, and 165,868,158 high-quality reads for Col-0, Cvi-0, and U112-3, respectively. The Q30 percentage of reads in each library was ≥88%. The reads from the three ecotypes were aligned to the *A. thaliana* reference genome (TAIR10) using the STAR universal RNA-seq alignment tool with default parameters. A total of 166,058,758, (95.93%), 174,167,848 (92.53%) and 165,868,158 (93.67%) quality-filtered reads were mapped to the reference genomes for Col-0, Cvi-0, and U112-3, respectively; ~8% of the reads remained unmapped (Table 1).

**Figure 6:**
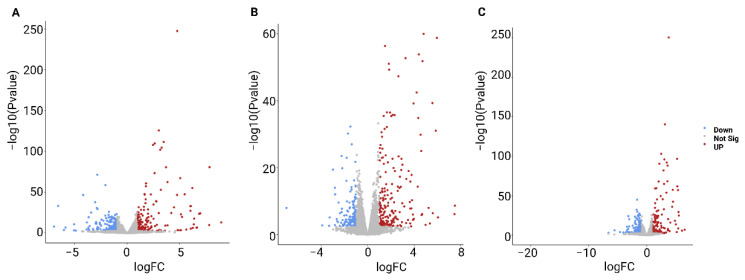
Volcano plot showing the differentially expressed genes (DEGs) from (**A**) Col-0, (**B**) Cvi-0, and (**C**) U112-3. Blue dots represent downregulated genes, and red dots represent upregulated genes; grey dots are not significantly up- or downregulated.

**Figure 7:**
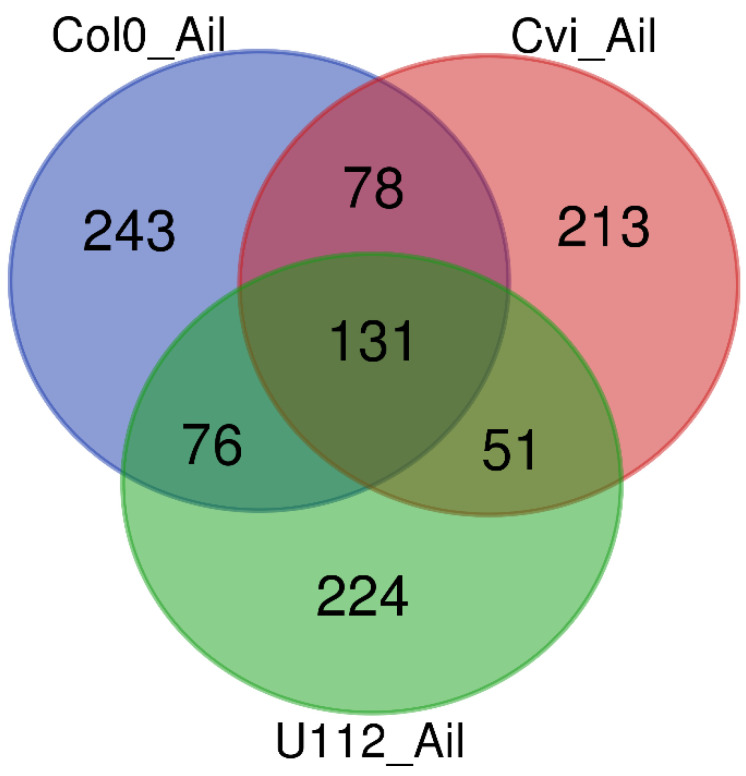
Venn diagram showing the number of differentially expressed genes obtained from ailanthone treatment with three Arabidopsis ecotypes, Col0, Cvi-0, and U112-3.

**Figure 8:**
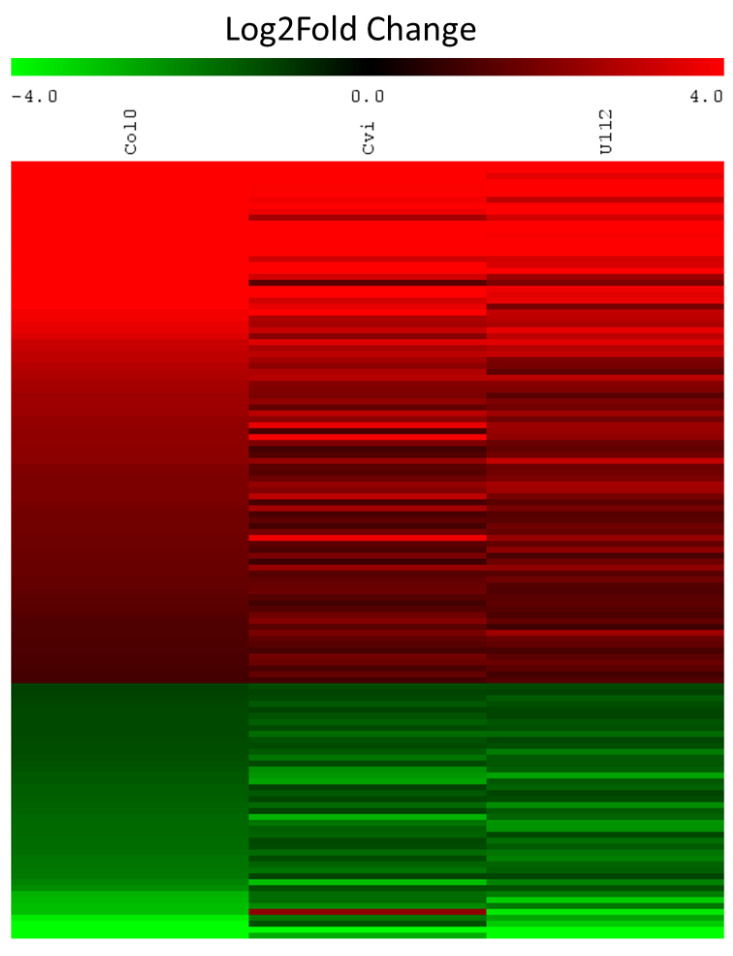
Heat map shows the expression pattern of the 131 common DEGs of Col-0, Cvi-0, and U112-3. The expression values are shown as log2 fold change.

In total, 528, 473, and 482 statistically significant differentially expressed genes (DEGs) were identified for Col-0, Cvi-0, and U112-3, respectively. The volcano plot in Figure 6 shows each ecotype’s total up- and downregulated genes based on −log10 (*p*-value) and log2 fold change. It includes 231, 266, 178 upregulated and 297, 207, and 304 downregulated genes. The Venn diagram comparing Col-0, Cvi-0, and U112-3 to identify DEGs that were shared between the three ecotypes is presented in Figure 7. The top 10 up- and downregulated DEGs shared among the three ecotypes are presented in Table 2. The heat map showing the expression profile of 131 common DEGs shared among the three ecotypes is shown in Figure 8.

### 2.7. Gene Ontology (GO) Enrichment Analysis

**Figure 9:**
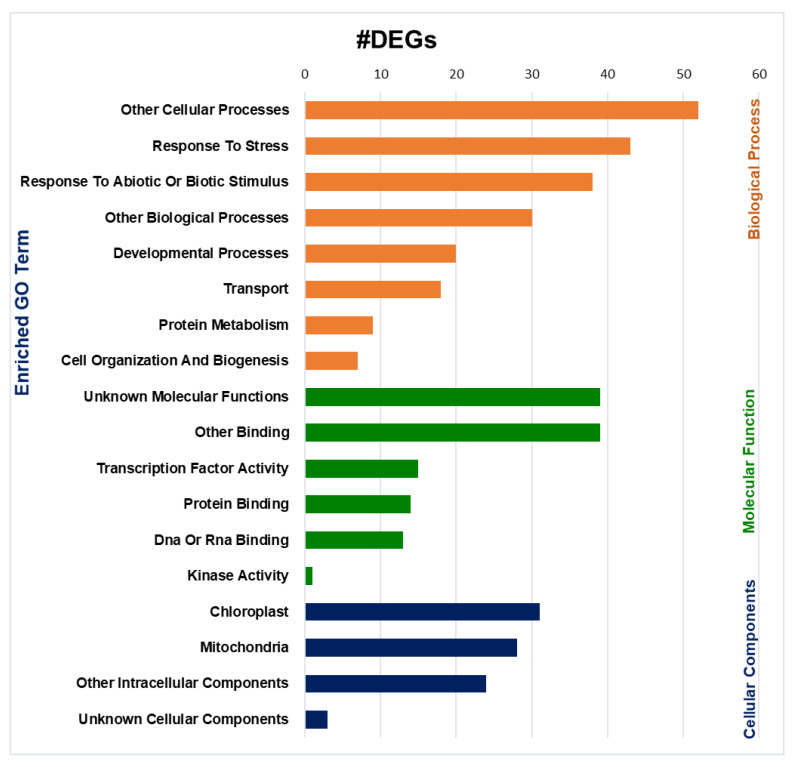
Enriched gene ontology terms from the classification of 131 DEGs shared by Col-0, Cvi-0, and U112-3 ecotypes.

GO enrichment analysis helps to understand DEG regulation under three important functional categories: biological processes, molecular functions, and cellular components. GO enrichment analysis of the 131 common DEGs from the three ecotypes Col-0, Cvi-0, and U112-3 revealed the functional categories enriched with ailanthone treatment. Significantly enriched biological processes with ailanthone treatment included several stress-response processes and plant-development processes. This includes response to stress (*n* = 43), response to abiotic or biotic stimulus (*n* = 38) and plant-developmental process (*n* = 20). Transcription factor activity was enriched with 15 DEGs under the molecular function. GO enrichment also revealed that chloroplasts carried 31 DEGs and mitochondria carried 28 DEGs under the cellular component category (Figure 9).

### 2.8. Pathway Enrichment Analysis

**Figure 10:**
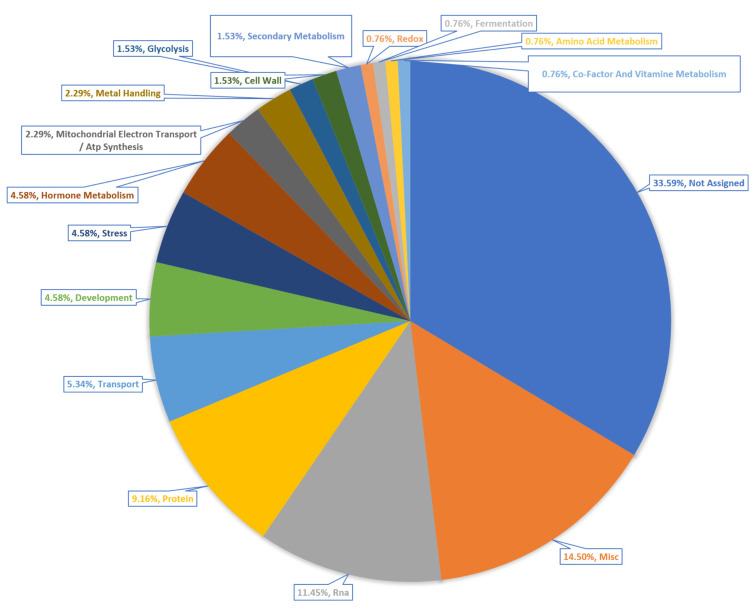
Enriched biological pathways from the 131 DEGs shared by Col-0, Cvi-0, and U112-3 ecotypes.

KEGG pathway enrichment analysis of the DEGs from each of the ecotypes showed different pathways enriched at different levels across the ecotypes. Pathway analysis of the 131 common DEGs with ailanthone treatment revealed several enriched pathways such as stress, development, hormonal metabolism, electron transport or ATP synthesis, glycolysis, cofactor, and vitamin metabolism. Overall, 44 DEGs were not assigned to any of the pathways (Figure 10). 

### 2.9. DEGs for Herbicides

**Figure 11:**
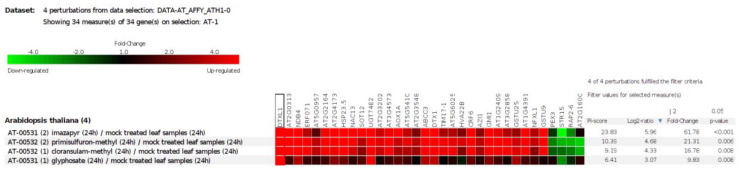
Common set of genes that were differentially expressed in the transcriptome of ailanthone-treated Col-0, Cvi-0, and U112-3 plants overlapping with the previously reported transcriptome profiles of herbicide-treated samples analyzed by using the Genevestigator database (accessed on 12 September 2022) (https://genevestigator.com/gv/).

We used Genevestigator to compare the expression of a common set of DEGs in these accessions with publicly available transcriptome databases of herbicide-treated samples; 34 genes showed a correlated expression pattern. Three genes were downregulated and 31 upregulated, which suggests that ailanthone treatment induced the expression of genes that were targeted by other herbicides (Figure 11).

**Figure 12:**
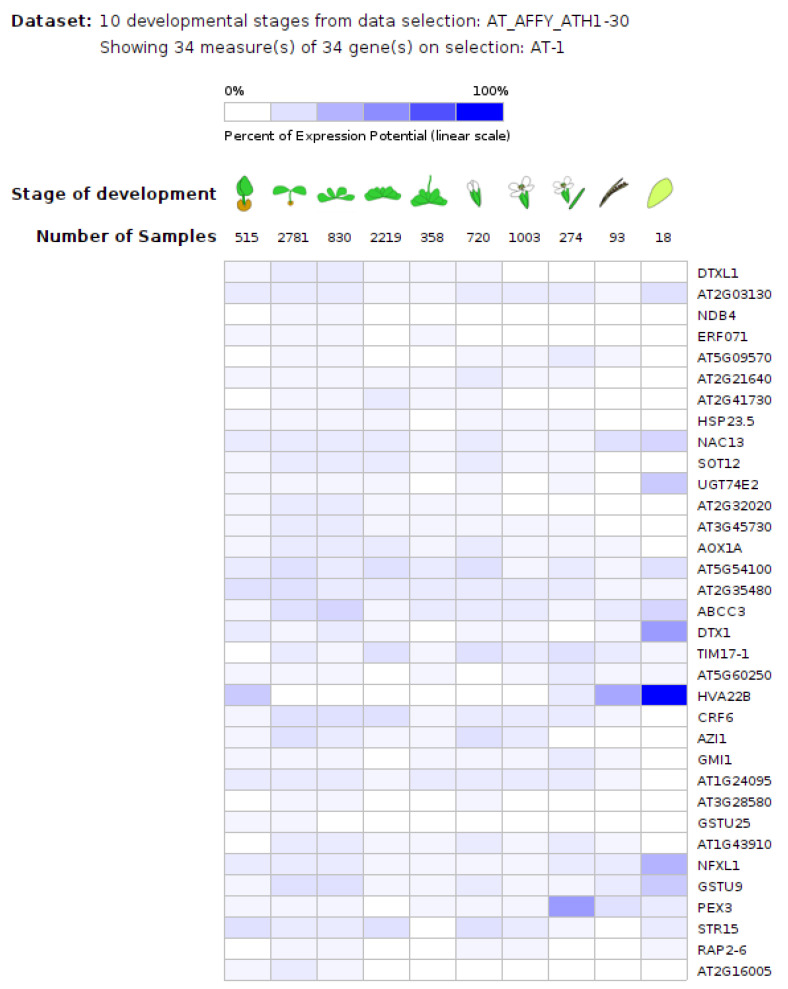
Developmental expression pattern analysis of a common set of genes that were differentially expressed in the transcriptome of ailanthone-treated Col-0, Cvi-0, and U112-3 samples using the Genevestigator database (accessed on 12 September 2022) (https://genevestigator.com/gv/).

The 34 common genes with an overlapped expression pattern in ailanthone- and herbicide-treated samples also shared a developmental expression pattern during plant growth. Genevestigator analysis showed the expression of these genes in seedlings, rosette leaves, flowers, silique, and seeds, thus indicating the ubiquitous nature of this expression (Figure 12).

**Figure 13:**
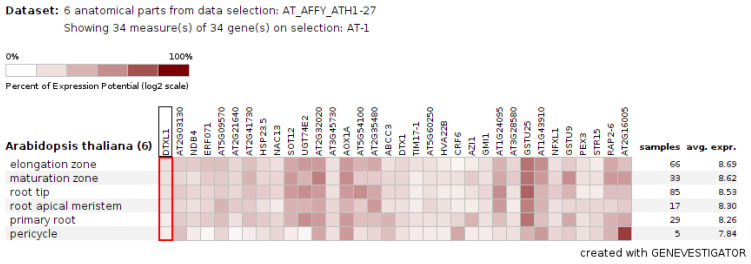
Root-tissue-specific expression pattern of the common set of genes (*n* = 34) that were differentially expressed in the transcriptome of ailanthone-treated Col-0, Cvi-0, and U112-3 samples analyzed by using the Genevestigator database (accessed on 12 September 2022) (https://genevestigator.com/gv/).

Because ailanthone-treated plants showed a strong inhibition of primary root growth and excessive accumulation of ROS, we analyzed the expression of the 34 common genes specifically in root tissues. Root-tissue-specific expression analysis with Genevestigator revealed that these 34 genes had a similar expression pattern in all root tissues, as in other organs (Figure 13).

## 3. Discussion

Ailanthone is a novel compound derived from the tree *A. altissima*. Ailanthone is an allopathic chemical and has a wide range of commercial values. It was isolated by Heisey in 1996 [7]. It has excellent drug-like characteristics in prostate cancer treatment and also works as a herbicide inhibiting the growth of other plants [8]. Historically, common salts and other metal salts were used for controlling weed plant growth. Still, most developed countries have banned the use of these salts because of their toxicity and persistence in soil and groundwater contamination. Plant-derived herbicides have overcome these disadvantages and are economical friendly [9,10,11]. In such a scenario, ailanthone is one of the best herbicides, but the molecular and metabolic nature of its activity is not yet characterized.

Here we used purified commercially available ailanthone and standardized the minimal concentration required for inhibiting plant growth. To determine the minimum concentration, we grew *A. thaliana* on a range of concentrations from 0.1 to 1 µM and determined that 0.5 µM was sufficient and able to inhibit plant growth (Figure 2). Ailanthone-treated seedlings showed herbicidal phenotypes, including reduced root growth, root volume, and rosette size. 

Basta is a commercially available herbicide belonging to glufosinate. It is used to control 80 types of weeds and serves as an excellent selection marker for transgenic lines [12,13]. Basta inhibits root growth and rosette size and induces chlorophyll bleaching. Ailanthone-treated plants also showed a similar range of phenotypes (Figure 1), so ailanthone could be a potential herbicidal chemical for a wide range of weed control once we identify and characterize its molecular nature regarding plant-growth inhibition. 

We chose three Arabidopsis accessions, Col-0, Cvi-0, and U112-3, because they show strong variation in growth and metabolism. Thus, we could investigate a common set of molecular signatures triggered by ailanthone treatment [14]. Col-0, Cvi-0, and U112-3 seeds were surface-sterilized and grown in 0.5 µM ailanthone along with mock controls, and seedling response was checked by analyzing root tips, root volume, forks, and crossings (Figure 3). Control-treated Cvi-0 plants exhibited a higher number of root tips than the other two accessions. Similar results were observed in earlier reports with respect to secondary growth in hypocotyls of these accessions [14]. As expected with normal herbicide treatments, the 0.5-µM ailanthone-treated Cvi-0 root-tip number was greatly reduced from 212 to 79, a reduction of 62%. Ailanthone-treated Col-0 and U112-3 seedlings also showed a reduction in the total root-tip number but to a lesser extent (50%), which suggests that Cvi-0 plants are more sensitive to ailanthone than Col-0 and U112-3 (Figure 3). Root forks and crossovers also showed a similar trend with ailanthone treatment in Col-0, Cvi-0, and U112-3. These results suggest that ailanthone inhibits root growth, thereby inducing stress on plant growth and development.

Proline accumulation is tightly linked with stress adaptation in plants. Increased proline levels in plants are inversely correlated with plant growth under stress [15]. In general, under normal osmotic conditions, proline levels remain very low; however, oxidative damage due to several stress factors spikes proline accumulation in plants. Elevated proline content in ailanthone treated Col-0, Cvi-0, and U112-3 seedlings indicated that these seedlings experienced stress in the presence of ailanthone, which resulted in reduced growth in a dose dependent manner (Figure 1, Figure 3 and Figure 4). When a metabolic disorder occurs in plants due to stress, this triggers oxidative destruction as a result of the excessive accumulation of ROS [16]. The over-accumulation of ROS leads to membrane damage and lipidic peroxidation, which can be monitored by 3,3′-diaminobenzidine (DAB) staining [17,18]. As mentioned previously, ailanthone treatment inhibits root-tip growth, possibly due to excessive accumulation of ROS. We checked this possibility by staining Col-0, Cvi-0, and U112-3 accessions with DAB and found a significant increase in DAB staining in the roots of ailanthone-treated samples as compared with control plants (Figure 5), which suggests that ailanthone induces stress similarly to other herbicides [19].

To obtain more information on the ailanthone effect on plant growth and development, we treated Col-0, Cvi-0, and U112-3 seedlings with 0.5 µM ailanthone along with mock treatment and performed global transcriptome analysis by RNA-seq. A total of 178,639,612, 187,148,924, and 178,521,414 raw reads were generated for Col-0, Cvi-0, and U112-3, respectively. The Q30 percentage of reads in each library was ≥88%. The reads from the 3 ecotypes were aligned to the *A. thaliana* reference genome (TAIR10) by using the STAR universal RNA-seq alignment tool with default parameters. A total of 166,058,758, (95.93%), 174,167,848 (92.53%), and 165,868,158 (93.67%) quality-filtered reads were mapped to the reference genomes for Col-0, Cvi-0, and U112-3, respectively; ~8% of the reads remained unmapped. Overall, 528, 473, and 482 statistically significant DEGs were identified for Col-0, Cvi-0, and U112-3, respectively: 231, 266, and 178 upregulated and 297, 207, and 304 downregulated, respectively. 

The three accessions shared 131 genes, which were differentially expressed with ailanthone treatment in comparison to controls (Figure 8): 82 were upregulated and 42 downregulated by at least twofold. To check the molecular nature and metabolic effect of ailanthone treatment, we compared these 131 common genes with the publicly available transcriptome database using Genvestigator (Figure 11, Figure 12 and Figure 13). In all, 34 of the 131 genes had a similar expression pattern when Arabidopsis seedlings were subjected to other herbicides such as imazapyr, primisulfuron-methyl, cloransulam-methyl, and glyphosate [20,21,22], which suggests that ailanthone treatment disturbs the metabolic pathways in a similar manner as other herbicides.

In corroboration with the ailanthone herbicide effect, two of the DEGs, *DTXL1* and *DTX1*, belonging to the multidrug and toxic compound extrusion or multi-antimicrobial extrusion (MATE) family [23], were upregulated more than 15- and 4.5-fold, respectively, in Col-0, Cvi-0, and U112-3 accessions. MATE genes encode efflux proteins, and their function is conserved in all kingdoms of life. The genes contain 12 transmembrane domains and participate in the export of various primary and secondary metabolites out of the cytosol by an electrochemical gradient mechanism. MATE proteins efflux and compartmentalize the metabolites when plants are challenged with disease and nutrient stress or on exposure to toxic compounds such as noxious or heavy metals, which therefore detoxifies their effect. These proteins are commonly called DETOXIFICATION (DTX) proteins in plants [24,25]. The Arabidopsis genome encodes 56 MATE genes and they have redundant function in detoxification of metabolites. The induction of MATE gene expression (*DTXL1* and *DTX1*) upon ailanthone treatment further strengthens the idea that ailanthone has a herbicidal activity in plants by modulating the primary and secondary metabolic pathways that are generally disturbed by stress and toxic compound treatment.

The ATP-dependent multidrug-resistant transporter ABCC3, also called multidrug resistance-associated protein 3 (MRP3) [26,27], was upregulated 4.8-fold in Col-0, Cvi-0, and U112-3 accessions with ailanthone treatment. Besides MRP3 being induced by heavy metals such as cadmium, nickel, arsenic, cobalt, and lead, herbicides can also strongly induce MRP3 transcription, thus implicating the ailanthone function in the MRP3 pathway [26,28]. Similar to the compartmentalization of toxic metabolites by MATE proteins, MRP3 proteins are also involved in vacuolar sequestration of toxic metabolites in cells. Analysis of a GUS-reporter-fused MRP3 promoter showed strong GUS expression in roots [26], which agrees with ailanthone-treated plant root defects and suggests that ailanthone treatment triggers MRP3 activity like other stresses. 

NA(P)H dehydrogenase B4 (*NDB4*) gene expression was upregulated >12-fold with ailanthone treatment in the three accessions. *NDB4* is one of the components of the non-phosphorylating alternative respiratory electron transport chain pathway in plants [24,29,30]. This alternative respiratory pathway is involved in preventing cellular damage when plants are exposed to biotic or abiotic stress [30]. Recently, the T-DNA insertion line or RNAi lines of *NDB4* were found to exhibit lower ROS formation, and plants showed altered leaf area and root:shoot ratios. *NDB4* RNAi lines performed better with salinity stress than did control plants, thus implicating an *NDB4* role in the stress response [29,30]. Moreover, T-DNA lines of *NDB2*, the homolog of *NDB4*, also showed better tolerance to salinity stress, whereas overexpressing *NDB2* plants were sensitive to salinity stress, which confirms that the NDB group of genes plays a key role in the stress response. NDB and alternative oxidase (*AOX*) genes genetically interact with and work in a similar pathway [29,30]. A single ndb2 mutant showed a reduction of 90% external NADH oxidation in isolated mitochondria. Overexpression of *NDB2* alone did not induce the NADH oxidation, but when combined with *AOX* overexpression, NADH oxidation was increased in isolated mitochondria. *AOX* expression was also increased 4.6-fold in ailanthone-treated Col-0, Cvi-0, and U112-3 accessions, which suggests that indeed ailanthone induces the NDB-mediated stress response in plants. These observations suggest that to cope with the stress response, plants trigger *NDB4* activity and promote alternative respiratory electron transport. Because ailanthone treatment induces *NDB4* and *AOX* expression, ailanthone may promote plant stress in a similar way as with other stresses.

Along with the induction of transporter gene expression in ailanthone-treated plants, two plant hormone metabolism genes for auxin and salicylic acid, uridine diphosphate glycosyltransferase 74E2 (UGT74E2) and azelaic-acid-induced 1 (AZI1), were upregulated 6.0- and 4.4-fold, respectively, with ailanthone treatment. Moreover, these genes were also induced with imazapyr, primisulfuron-methyl, cloransulam-methyl, and glyphosate herbicide treatment and their function has been implicated in defense mechanisms [31,32]. UGT74E2 controls auxin homeostasis by regulating the biosynthesis pathway of indole-3-butyric acid [32]. H_2_O_2_ strongly induced UGT74E2 expression, so its function has been implicated in the ROS pathway. In agreement with UGT74E2 function, ailanthone-treated roots were also strongly stained with DAB, a reporter of ROS activity, which suggests that ailanthone-induced ROS accumulation is possibly due to upregulation of UGT74E2. AZ1 is involved in systemic immunity response, when plants are attacked by a pathogen or azelaic acid [31,33,34]. AZ1 works in the salicylic acid pathway and its localization to chloroplasts is increased during the systemic immunity response. However, recently, a natural allelic variation in AZ1 was found to fine-tune root growth under nutrient-limited conditions, specifically zinc depletion [35], which points to the dual role of AZ1 in the immune response and adjustment of plant growth to nutrient conditions. Taken together, these results suggest that ailanthone triggers a stress response in multiple pathways similar to other herbicides. 

## 4. Materials and Methods

### 4.1. Collection of Plant Materials and Root Trait Data

A collection of *A. thaliana* accessions (Col-0, Cvi-0, and U112-3) were ordered from the Arabidopsis Biological Resources Center (ABRC) at Ohio State University. All *A. thaliana* plants were grown under constant conditions (25 °C) in a Percival grow chamber. The seeds were sown on square Petri plates on Murashige and Skoog (MS) medium. The seeds were sown, sealed, and incubated for 72 h at 4 °C. The plants were grown for 1 week on control media, then transferred onto treatment and control plates using 50 mL MS media and adding the treatment (0.5 µM ailanthone). The plants were marked where the root growth stopped and then grown on control and treatment plates for an additional 7 days. The plants grown on control and treatment plates were used for the physiological studies and transcriptome analysis. Root-trait data such as overall root length, number of root hairs, tips, crossing, and forks were measured by using WinRhizo (http://regentinstruments.com, accessed on 12 September 2022).

### 4.2. Reactive Oxygen Species (ROS) Assay with DAB Staining

ROS accumulate when plants are under various biotic and abiotic stresses. Excessive ROS levels cause oxidative damage to proteins, DNA, and lipids. ROS also act as signaling molecules to regulate development and stress responses [36]. H_2_O_2_ was detected in mature Arabidopsis rosette leaves and root tissue by staining with 3, 3′-diaminobenzidine (DAB) by vacuum infiltration. DAB is oxidized by H_2_O_2_ in the presence of heme-containing proteins, such as peroxidases, to generate a dark brown precipitate. This precipitate was used as a stain to detect the presence and distribution of H_2_O_2_ in plant cells [37]. The dark-brown precipitate was then visualized, and pictures of control and treatment plants were taken to observe the localization of H_2_O_2_ in leaf and root tissue.

### 4.3. Total RNA Isolation and RNA Sequencing

A whole-plant sample was collected from the control and treatment plates with three biological replicates for each condition. Total RNA was isolated from these whole plant tissues of the *A. thaliana* ecotypes, Col-0, Cvi-0, and U112-3, using the E.Z.N.A. Plant RNA Kit (Omega Bio-tek, Norcross, GA, USA). The quality of the total RNA was assessed using agarose gel electrophoresis and a bioanalyzer (Agilent, Santa Clara, CA, USA). The quantity of the RNA was estimated by using a qubit fluorimeter (Invitrogen, Waltham, MA, USA). An RNA-seq library was prepared for each of the ecotypes separately by using NEBNext Ultra II RNA Library Prep Kit for Illumina with NEBNext Poly (A) mRNA Magnetic Isolation Module (NEB, Ipswich, MA, USA). The RNA-seq library quality and quantity were assessed by using a Bioanalyzer (Agilent, Santa Clara, CA, USA) and qubit fluorimeter (Invitrogen, Waltham, MA, USA). The library was subjected to paired-end sequencing (2 × 75 bp) with the Illumina NextSeq 500 platform (Illumina, San Diego, CA, USA).

### 4.4. Transcriptome Analysis

The generated image files in the BCL format from NextSeq 500 were converted to FASTQ with 2 × 75 bp reads by using the bcl2fastq tool (Illumina, San Diego, CA, USA). The quality of raw reads was analyzed by checking the adapter content, GC content distribution, and average base quality score of the reads by using the fastqc tool (https://www.bioinformatics.babraham.ac.uk/projects/fastqc/, accessed on 12 September 2022). The sequencing adapters and low-quality reads (Phred score QV < 30) were removed by using the read trimming tool Trimmomatic [38]. The quality-filtered reads were mapped to the *A. thaliana* reference genome TAIR 10 (https://phytozome-next.jgi.doe.gov/info/Athaliana_Araport11, accessed on 12 September 2022) using the STAR RNA-Seq aligner [39] to generate BAM alignment. The read count table was generated from the BAM alignment file and general feature format (GFF) of genome annotation using the HTSeq R package [40]. The differentially expressed genes (DEGs) among the three experimental pair-wise combinations were identified using the DESeq2 R package [41]. The DEGs were filtered based on the minimum log2 fold change = 1 and *p* < 0.05. Gene annotations were obtained by using Omicsbox (https://www.biobam.com/omicsbox/, accessed on 12 September 2022). Gene ontology (GO) enrichment analysis was performed with ShinyGO. Pathway analysis of DEGs was performed using the Kyoto Encyclopedia of Genes and Genomes (KEGG) pathway database with ShinyGO (http://bioinformatics.sdstate.edu/go/, accessed on 12 September 2022). Gene Investigator (https://genevestigator.com/, accessed on 12 September 2022) was used to identify the gene expression studies involving the herbicides that were previously reported.

## 5. Conclusions

We analyzed the physiological and transcriptomic response of ailanthone in three ecotypes of Arabidopsis. We identified 131 common DEGs that were induced in common in all three ecotypes. These DEGs included 34 genes whose activity was directly correlated with the herbicide response previously reported by four independent gene-expression studies. We identified several important genes and biological pathways that were altered in response to ailanthone in Arabidopsis. These results suggest that ailanthone triggers a stress response in multiple pathways similar to other herbicides. 

## Figures and Tables

**Table 1:** Summary of RNA sequencing and genome mapping.

Sample ID	Total Number of Raw Reads	Total Number of Quality Filtered Reads	Mapping Percentage
Col0_Ail_1	29,168,346	27,177,582	95.8
Col0_Ail_2	34,322,102	31,984,630	96.4
Col0_Ail_3	30,841,378	28,664,400	95.8
Col0_CTRL_1	27,687,690	25,789,876	96. 8
Col0_CTRL_2	30,188,048	28,110,900	94.9
Col0_CTRL_3	26,432,048	24,331,370	95.8
Cvi_Ail_1	32,780,320	30,590,034	89.9
Cvi_Ail_2	29,514,950	27,302,574	94.2
Cvi_Ail_3	33,177,798	30,919,340	88.9
Cvi_CTRL_1	31,355,394	29,189,452	94.6
Cvi_CTRL_2	30,062,314	28,001,312	94.7
Cvi_CTRL_3	30,258,148	28,165,136	92.6
U112_Ail_1	32,870,152	30,580,308	95.8
U112_Ail_2	33,209,324	30,938,080	94.8
U112_Ail_3	25,723,286	23,922,282	91.1
U112_CTRL_1	29,311,232	27,242,308	93.8
U112_CTRL_2	31,819,904	29,597,306	90.7
U112_CTRL_3	25,587,516	23,587,874	95.6

**Table 2:** Common DEGs identified across 3 ecotypes in response to Ailanthone.

Gene ID	Annotation	Col-0	Cvi-0	U112-3	Up/Downregulated
AT5G54550	hypothetical protein (DUF295)	8.95	7.51	5.00	UP
AT2G04050	MATE efflux family protein	7.83	4.61	5.22	UP
AT2G03130	Ribosomal protein L12/ATP-dependent Clp protease adaptor protein ClpS family protein	7.80	4.69	3.61	UP
AT3G58150	Optic atrophy 3 protein (OPA3)	6.96	5.58	4.37	UP
AT2G42065	DnaJ domain protein	6.94	3.95	5.42	UP
AT3G54530	hypothetical protein	6.85	4.55	5.52	UP
AT5G55150	F-box SKIP23-like protein (DUF295)	6.65	3.82	3.02	UP
AT5G54560	hypothetical protein (DUF295)	6.60	6.05	6.05	UP
AT2G20800	NAD(P)H dehydrogenase B4 (*NDB4*)	6.34	3.76	4.46	UP
AT5G54450	hypothetical protein (DUF295)	6.24	2.60	3.30	UP
AT5G36130	Cytochrome P450 superfamily protein	−2.08	−2.99	−2.05	DOWN
AT4G40020	Myosin heavy chain-related protein	−2.24	−1.23	−1.26	DOWN
AT4G31940	cytochrome P450, family 82, subfamily C, polypeptide 4 (CYP82C4)	−2.81	−1.70	−1.98	DOWN
AT5G23990	ferric reduction oxidase 5 (FRO5)	−2.89	−1.69	−3.21	DOWN
AT1G47400	hypothetical protein	−2.99	−2.14	−1.88	DOWN
AT2G29350	senescence-associated gene 13 (SAG13)	−3.01	−2.21	−3.64	DOWN
AT4G09110	RING/U-box superfamily protein	−3.70	−1.85	−2.62	DOWN
AT2G30750	cytochrome P450 family 71 polypeptide (CYP71A12)	−4.19	−1.28	−3.19	DOWN
AT5G38910	RmlC-like cupins superfamily protein	−6.57	−7.00	−4.62	DOWN
AT3G12900	2-oxoglutarate (2OG) and Fe(II)-dependent oxygenase superfamily protein	−6.96	−2.72	−4.05	DOWN

## Data Availability

The raw paired-end sequencing reads from the current study is available at NCBI under the BioProject accession number PRJNA870106.
